# Neutrophils as a potential therapeutic target in Alzheimer’s disease

**DOI:** 10.3389/fimmu.2023.1123149

**Published:** 2023-03-03

**Authors:** Michelle L. Aries, Tiffany Hensley-McBain

**Affiliations:** McLaughlin Research Institute, Great Falls, MT, United States

**Keywords:** Alzheimer’s disease, neutrophils, mouse models, human studies, transcriptomics, inflammation, neuroinflammation, ApoE4

## Abstract

Alzheimer’s disease (AD) is the leading cause of dementia in the United States. Sporadic or late-onset AD remains incompletely understood, with age as the current greatest risk factor. Inflammation in general and neutrophils, a potent mediator of inflammation, have been shown to exacerbate AD associated dementia. This review explores the latest research on neutrophils in AD mouse models and in human cohort studies and discusses current gaps in research and needs for future studies. AD mouse models have shown neutrophil chemotactic migration towards amyloid beta plaques in the brain. Capillary blood flow stalling decreases blood perfusion to associated brain regions and mouse studies have demonstrated that anti-Ly6G antibodies lead to a decrease in capillary blood flow stalling and memory improvement. Several recent transcriptomic studies of blood and brain tissue from persons with AD have shown an upregulation in neutrophil-related genes, and studies have demonstrated neutrophil involvement in brain capillary adhesion, blood brain barrier breaching, myeloperoxidase release, and the propensity for neutrophil extracellular trap release in AD. Neutrophil-derived inflammation and regulation are a potential potent novel therapeutic target for AD progression. Future studies should further investigate neutrophil functionality in AD. In addition, other aspects of AD that may impact neutrophils including the microbiome and the *APOE4* allele should be studied.

## 1 Introduction

**Figure 1:**
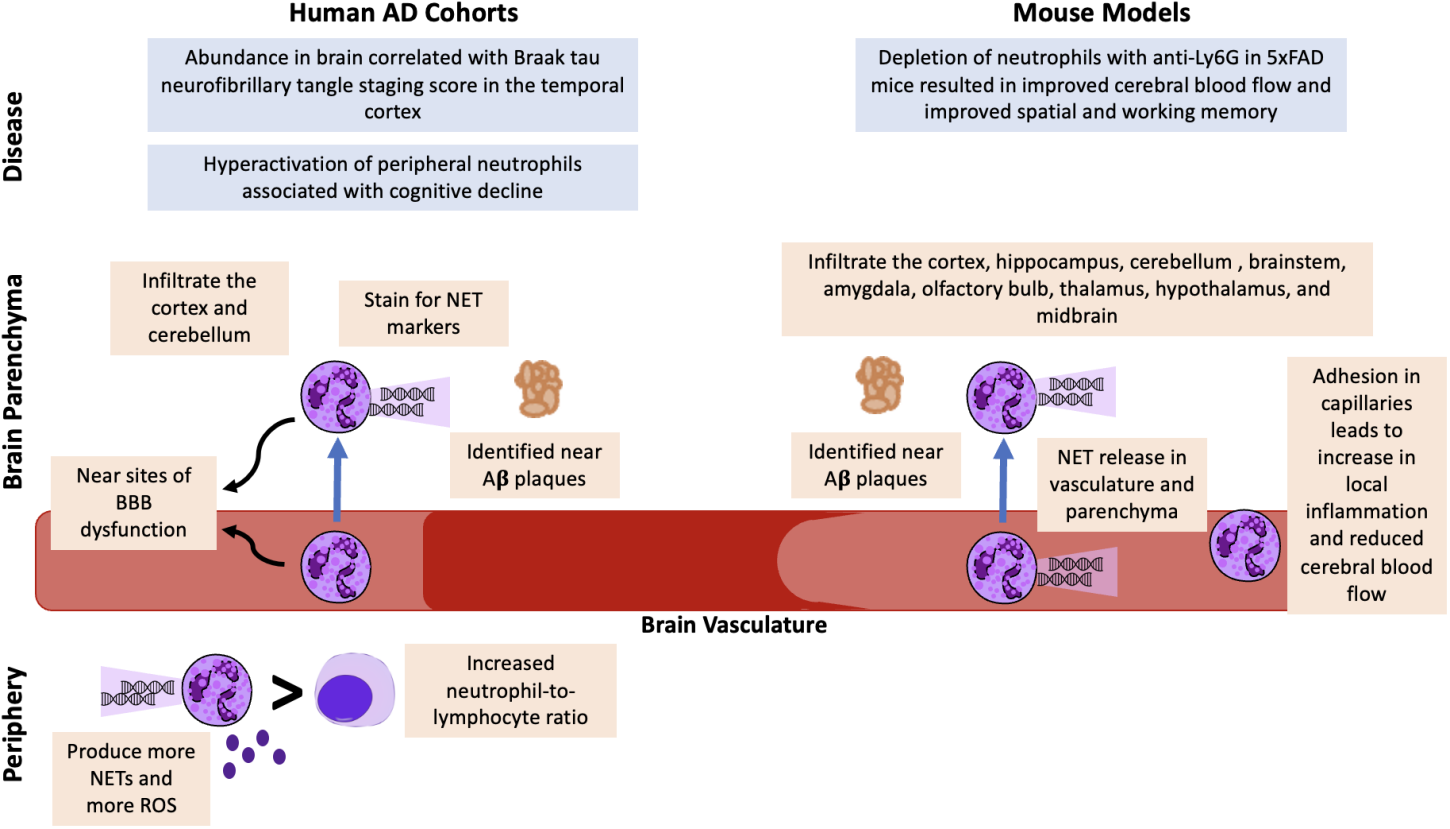
Potential Neutrophil Involvement in AD from Human AD Cohorts and AD Mouse Model Studies. Neutrophils have been identified in the brain vasculature and parenchyma in human cohorts and mouse models of AD. In both humans and mice, neutrophils are found near Aβ plaques and stain for NET markers. Neutrophils have been associated with BBB dysfunction, and mouse models suggest they also contribute to reduced cerebral blood flow. An increased blood neutrophil-to-lymphocyte ratio and increased neutrophil activation, NET release, and ROS release have been identified as potential peripheral markers of AD. Finally, correlative analyses in humans have demonstrated associations with brain neutrophils and Braak staging and hyperactivation of peripheral neutrophils associated with cognitive decline. Mechanistic studies in mice demonstrated that depleting neutrophils resulted in improved cerebral blood flow and improvement in behavioral tests.

Alzheimer’s disease (AD) is the most common cause of dementia and the 6^th^ leading cause of death in the United States (1). More than 6.5 million people in the United States are estimated to be living with AD (1), which is characterized by amyloid beta (Aβ) plaques and neurofibrillary tau protein-containing tangles. While the amyloid hypothesis suggests that misfolded Aβ is the toxic and causative agent of AD, the field has shifted toward investigating other potential causes and therapeutic targets (2, 3). Age remains the greatest risk factor for AD, yet there is a need to delineate contributions of additional factors and how they synergize with age. Chronic systemic inflammation and immune activation are associated with AD pathogenesis, and neuroinflammation mediated by astrocytes and glial cells is observed in the brain prior to the onset of cognitive decline (4–6). Peripheral inflammation results in non-resident immune cells, including monocytes, T cells, and neutrophils, crossing the blood brain barrier (BBB) to contribute to damage and cognitive decline (7). The contribution of the immune system to AD is now so widely accepted that the largest single category of drugs in clinical trials for AD in 2022 are those targeting inflammation or the immune system (8). Despite the central role of neutrophils in inflammatory responses, neutrophils have traditionally been understudied in AD. However, recent evidence suggests they contribute to neuroinflammation, neurodegeneration, and cognitive decline, and their typical residence outside of the brain makes them an accessible and promising therapeutic target for AD. In this review, we focus on neutrophil involvement in AD, most of which has been investigated in mouse models. **Figure 1** is an overview of potential neutrophil involvement in AD based on neutrophil data from previous human AD cohorts and mouse AD model studies. **Table 1** contains details on the mouse model, microscopy imaging technique, region of the brain where neutrophils were found and the antibodies and/or stains used for visualization. We also provide a summary of neutrophil studies in human cohorts and tissues, and review multiple transcriptomic studies that have indicated neutrophil-related signatures as top pathways altered in AD. The evidence is strong that neutrophils are dysregulated in AD, suggesting they may be a therapeutic target to alleviate disease. However, the lack of studies on neutrophil functionality in AD, their relationship to genetic risk factors, sex, and age, and studies mechanistically linking human neutrophils to disease are needed to fully assess their therapeutic utility in AD.

**Table 1:** Alzheimer’s Disease Mouse Model Studies.

Reference	Mouse Model	Age in Months	Neutrophil Detection	Findings
**Baik et al.** (9)	5xFAD TG6799; B6SJL-Tg [APPSwF1Lon, PSEN*M146L* L286V]6799Vas/J stock no. 006554	8	Allophycocyanin-conjugated Ly6G (GR-1)	Neutrophils were observed in the frontal cortex using flow cytometry.
		9 – 13	0.12 mg/kg Ly6C/G (GR-1) was IV injected right before imaging in the femoral vein	2-photon *in vivo* microscopy found neutrophils in the parenchyma that originated from the blood.
**Cruz Hernández et al.** (10)	APP/PS1 B6.Cg-Tg(APPSwe,PSEN1dE9)85Dbo/J; MMRRC stock no. 034832-JAX	11 – 13	anti-CD45, anti-CD11b, anti-Ly6G	Neutrophil concentration in the blood after anti-Ly6G treatment was monitored using flow cytometry. Neutrophil concentration decreased six hours after treatment.
			Anti-Ly6G Alexa488 (0.1 mg/kg) was IV injected right before imaging in the tail vein	2-photon *in vivo* microscopy found neutrophils in the cerebral cortex parenchyma. They were observed to migrate from the blood.
	5xFAD B6SJL-Tg(APPSwF1Lon,PSEN1*M146L*L286V)6799Vas/Mmjax; MMRRC Stock no. 34840-JAX	5 – 6		
**Zenaro et al.** (11)	5xFAD APP with Swedish, Florida, and London and PS1 with M146L, L286V And 3xTg-AD PS1(M146V), ßAPP (Swedish) and tau (P301L) And 3xTg-AD PS1(M146V), ßAPP (Swedish) and tau (P301L) with Itgal^-/-^	2 – 8 and 4 – 10	anti-CD45, anti-CD11b, anti-Ly6G, anti-Ly6G	Whole brain homogenate was used to quantify neutrophil concentration by flow cytometry. The concentration of neutrophils was decreased in 3xTg-AD Itgal^-/-^ and mice treated with an anti-Ly6G antibody.
			CMTPX or CMAC	2-photon *in vivo* microscopy found neutrophils in the cortex and parenchyma. Neutrophils were observed migrating directional into the brain with noticeable velocity or with low motility and swarm behavior.
	5xFAD APP with Swedish, Florida, and London and PS1 with M146L, L286V And 3xTg-AD PS1(M146V), ßAPP (Swedish) and tau (P301L)	2 – 8 and 4 – 10	Ly6G and CD45 (fluorescent)	Confocal microscopy discovered neutrophils in the choroid plexus, hippocampus, and the vessels of the meninges and cortex. They were observed to migrate from the blood.
**Smyth et al.** (12)	APP/PS1 (APPswe,PSEN1dE9)85Dbo, MMRRC stock No:34832-JAX	4 and 12	MPO, S100A8, and CD66B	An increase in neutrophils in AD brains was observed using Zeiss Axio Imager and an automated fluorescence microscope.
**Kong et al.** (13)	3xTG B6;129-Psen1tm1MpmTg(APPSwe,tauP301L) 1Lfa/Mmjax	12	Ga-PEG-cFLFLFK	Increased neutrophil infiltration in the brains of Tg mice was observed using PET Imaging.

### 1.1 Alzheimer’s disease overview

AD is a neurodegenerative disease resulting in impairment of communication, memory, and the ability to perform daily living tasks (1, 8). Women make up almost two-thirds of all AD patients (1). Sex and age studies have demonstrated that women are twice as likely to have AD as men even at the age of 45 (1). The percentage of deaths caused by AD from 2000 to 2019 increased by 145%, whereas stroke and heart disease decreased by 10.5% and 7.3%, respectively (1). It is projected that the number of people living with AD will continue to rise (1). Early onset or familial AD typically presents before the age of 65 and is caused by autosomal dominant mutations in the amyloid precursor gene (APP) or the complexes involved in amyloid cleavage, presenilin 1 and 2. However, 99% of cases occur later in life (> 65 years of age), and the interconnected mechanisms involved in sporadic or late-onset AD (LOAD) remain incompletely understood (14). Previous studies have indicated that Aβ plaque deposits, phosphorylation of tau, tau tangles, immune dysfunction, chronic inflammation, BBB breakdown, vascular dysfunction, and neuronal death could begin to cause damage decades before symptoms are noticed or a diagnosis is made (1, 7, 15). Historically, AD drugs have tried to target Aβ fibril and plaque accumulation and tau tangles, as they are not only pathological hallmarks of AD, but studies have linked them mechanistically to memory, cognitive, and physical dysfunction (1, 3). Treatments aimed at neuroinflammation or increasing BBB integrity are more recently being studied (8). A combination of treatments for preventing or decreasing pathological contributors to AD, such Aβ plaques and tau tangles, while decreasing chronic inflammation and increasing BBB integrity could be instrumental in slowing the progression of AD. Since damage occurs before the onset of noticeable symptoms, methods for early detection are also crucial to prevent damage, and stabilize or improve memory, cognition, and physical function.

### 1.2 Alzheimer’s disease mouse models used in neutrophil studies

**Table 1** summarizes the mouse studies that have assessed neutrophils in AD and includes the mouse model description and strain number, if available. Each of the models that have been used to study neutrophils in AD overexpress transgenic human *amyloid precursor protein* (*APP*) under the mouse Thy1 or PrP promotor to drive expression in central nervous system (CNS) neurons. They all represent familial AD models since they also contain familial mutations associated with AD in the human *APP* transgene. Specifically, 5xFAD mice include the London mutation (V717I), the Swedish mutation (K595N/M596L), and the Florida mutation (I716V), while 3xTg-AD and APP/PS1 mice contain only the Swedish mutation (16–18). These models also express transgenic mutant human *presenilin 1* (*PS1)* under the same promotors, which encodes for the catalytic subunit of the γ-secretase responsible for Aβ production. One commonly used model, 3xTg-AD, also includes human transgenic *microtubule associated protein tau* (*MAPT*) with the P301L mutation in addition to mutant human *APP* and *PS1*. All of these models overexpress Aβ in the CNS and develop diffuse Aβ plaques starting as early as 2-3 months of age (19). Memory tasks in these APP mouse models to assess AD-like progression temporally follow the progression of AD in humans, with the earliest impairments demonstrated in spatial working memory through a Barnes maze or Morris water maze task (19). In these tasks, mice use visuospatial cues to find a target, such as an escape hole in the Barnes maze or a raised platform in the water maze. Deficits in recognition memory present later and have been demonstrated with the novel object recognition task, which measures the animal’s innate exploratory behavior. These models are best representative of the pathophysiology of familial AD and have multiple drawbacks, including the nonphysiological, overexpression of Aβ, potential disruption of endogenous genes by the transgenes, and lack relevance to LOAD. Recently, there have been new developments in attempts to generate better LOAD models that include risk genes for AD, such as *APOE* and *TREM2 (* 20). However, neutrophils have yet to be examined in LOAD models or in the context of LOAD genetic risk factors, an important gap in understanding neutrophils in AD, as discussed below.

## 2 Neutrophils in normal physiology and disease

Neutrophils are formed in the bone marrow from myeloid precursors and are the most abundant leukocyte in humans and a dominant leukocyte population in mice (21). Neutrophils are critical in containing invading pathogens and have been widely studied for their antibacterial responses until recently they were mainly viewed as bacteria eaters (22). However, roles for neutrophils in antiviral responses, tissue repair, and mediating sterile inflammation have become evident. Neutrophils contain pathogens *via* several mechanisms including 1) phagocytosis, 2) the release of antimicrobial molecules through degranulation, and 3) containment and killing of pathogens *via* release of nuclear DNA, termed neutrophil extracellular traps (NETs) (22). While neutrophil inflammatory responses are beneficial in controlling infection, they can also cause unintended tissue damage due their release of antimicrobial peptides, lytic enzymes meant to degrade extracellular matrix, and reactive oxygen species (ROS) (23). As such, neutrophils are known contributors to many inflammatory diseases, including inflammatory bowel disease, rheumatoid arthritis, diabetes, and cardiovascular disease (24). Recent evidence suggests neutrophils contribute to inflammation and disease progression in AD (9–13, 25–29). Aging is accompanied by low-grade systemic inflammation and dysregulation of the immune system, termed inflammaging (4), and neutrophil activation and alterations in cell death mechanisms may synergize with age to promote inflammation in AD. Indeed, neutrophils isolated from elderly individuals produced more ROS and had elevated CD11b, an adhesion molecule (30).

Neutrophils are generally short-lived due to their propensity to undergo preprogrammed homeostatic apoptosis, with findings from studies investigating neutrophil lifespan *in vivo* ranging from 8 hours to 5 days in humans and less than 1 day in mice (31–33). The least inflammatory mechanism of neutrophil clearance from tissues is caspase-3 mediated apoptosis followed by engulfment by macrophages (21, 32, 34). Neutrophil activation and lifespan is impacted by cytokine signaling and interactions with microbes and their products in the environment (35, 36). Conditions that delay or prevent neutrophil apoptosis can result in neutrophilia or secondary necrosis, a more inflammatory mechanism of cell death that releases intracellular contents, which can then cause host tissue damage. Delayed neutrophil apoptosis has been observed in multiple inflammatory diseases including acute respiratory disease syndrome, chronic pulmonary obstructive disease, cardiovascular disease, rheumatoid arthritis, and cystic fibrosis (37). However, the role of neutrophil lifespan perturbations in neurodegenerative diseases, including AD, has yet to be examined.

Finally, it should be mentioned that in addition to pro-inflammatory functions and contributions to resolution of inflammation and tissue repair, neutrophils can also suppress immune responses by acting as granulocytic myeloid derived suppressor cells (MDSCs). MDSCs arise from the myeloid lineage and impede both innate and adaptive immunity (38), and granulocytic MDSCs inhibit activation and expansion of T cells. While some studies suggest there may be a role for granulocytic MDSCs, particularly in the early stages of the disease, there are very few studies that have examined these cells in AD and no studies have assessed them in brain tissue of persons with AD or from AD mouse models (39).

### 2.1 Neutrophils in mouse models of Alzheimer’s disease

AD mouse models have been used to study the progression, prognosis, nuances of different pathophysiological mechanisms, novel therapeutic targets, and treatment for AD (19, 20). AD mouse models are a useful way to study the pathology of inflammation associated molecules and evaluate potential therapeutics (4). As discussed previously, each of the mouse models used to study neutrophils in AD have been familial AD models, with mutant human transgenes for *APP, PS1*, and in some cases *MAPT* (**Table 1**). Of note, there are important differences between mouse and human neutrophils, including blood frequencies, production of defensin molecules, receptor expression, morphology, signaling, and granule protein contents, so conclusions connecting neutrophil contributions to disease should be restricted to mouse neutrophils until confirmed in human neutrophils (40, 41). Mouse models of humanized neutrophils are lacking, with only one model reported to develop human neutrophils and no studies investigating humanized neutrophils in mouse models of disease (42). However, studies of mouse neutrophils in mouse models provide the foundation for investigating mechanisms in human blood and tissue samples and in *in vitro* systems. Studies investigating neutrophils in mice predominantly utilize either myeloperoxidase (MPO), which is often used as a surrogate neutrophil marker in both mouse and human studies, or Ly6G, which is a nearly exclusive neutrophil marker in mice with no known human equivalent (43, 44). Mouse model studies have shown that neutrophils exhibit migration along a chemotactic gradient towards Aβ plaques in the brain and migrate across the blood brain barrier (BBB) (7, 9–11, 13). Neutrophils have a higher meandering index and velocity in AD mice than their wild-type (WT) counter parts and have been shown to be in higher concentrations not only in brain-associated capillaries, but across the BBB as well (9, 10). Previous studies have demonstrated neutrophil accumulation at adhesion sites in brain capillaries, especially at locations of low vascular endothelial (VE) cadherin expression and breaching the BBB surrounding adhesion sites in AD mouse models, which was not observed in their age and sex-matched wild-type counterparts (7, 9–11). Increased expression of vascular cell adhesion molecule-1 (VCAM-1), P-selection, E-selectin, intercellular adhesion molecule-1 (ICAM-1) was found in the vessels of the cortex and meninges of 5xFAD mice as young as 4 months old (11). Neutrophils have been observed in multiple brain regions in AD models, including the cortex, hippocampus, cerebellum, brainstem, vessels of meninges, choroid plexus, amygdala, midbrain, hypothalamus, thalamus, and olfactory bulb (7, 10–13). Neutrophils are found near Aß plaques, are known to release NETs, and associate with tissue damage in AD models (11–13, 26). Increased NET release by neutrophils is observed in AD and has been proposed to be one of the possible mechanisms for neutrophil BBB breaching and neuronal damage (7, 11, 45). Importantly, MPO deficient AD model mice demonstrated better cognitive outcomes and reduced inflammation (26).

Neutrophil adhesion plays a significant role in AD pathology, as adhesion not only leads to neutrophil transport across the BBB and a local increase in inflammation, but could possibly result in chronic vascular permeability, and possible BBB damage (7, 9–12). Moreover, neutrophil accumulation has also been associated with capillary blood flow (CBF) stalling, which decreases blood perfusion in neighboring brain regions and increases cognitive dysfunction in mouse models (9, 10, 12). Mouse CBF networks demonstrated high similarity to CBF networks in humans, suggesting that this may also be a mechanism of AD pathogenesis in humans (10, 12).

Multiple studies have demonstrated that decreasing neutrophil accumulation and adhesion with neutrophil-targeting antibodies or with the use of LFA-1 deficient mice, decreases CBF stalling and increases spatial short-term memory (10, 11, 46). Moreover, early treatment of AD model mice, by neutrophil depletion or adhesion disruption (LFA-1 null mice), was shown to increase memory and act as a protectant for memory loss in the future, as young mice treated for a month showed memory improvement months after the treatment ended (11). These studies suggest neutrophils are a potential contributor to AD pathogenesis, and additional studies in mice are needed to elucidate their function, phenotype, and mechanisms of dysregulation.

### 2.2 Neutrophils in human cohorts of persons with Alzheimer’s disease

Studies investigating human cohorts have corroborated mouse model findings and have provided evidence that neutrophils have a role in AD pathogenesis. Transcriptional analyses of brain tissue across multiple studies have continually revealed a significant increase in differentially expressed genes (DEGs) related to neutrophil signaling pathways in AD patients compared with controls (29, 47–52). In fact, in several studies neutrophil DEGs were among the strongest contributors to differential signals in peripheral and brain transcripts (29, 49, 53). These studies revealed increased adhesion and cell surface interaction transcripts coupled with decreases in immature neutrophil biomarkers (29, 47, 49). Increased proinflammatory pathways in general are also common among these AD transcriptomic studies (29, 47, 49, 52), and these inflammation-related DEGs correlate most strongly with neutrophils (29). These neutrophil signatures also associate with AD progression across multiple studies (12, 25, 47, 54, 55).

It is now well established that neutrophils infiltrate the brain during AD (7, 11, 12, 29). Studies that investigated specific brain regions identified neutrophils in the temporal cortex, and hippocampus, and cerebellum of persons with AD in both the parenchyma and vasculature (11, 12, 29). Neutrophils in brains from persons with AD were observed near or at Aβ plaques, were more likely to stain for NET-associated markers, and were found near sites of BBB dysfunction (11, 12, 54, 55). Neutrophils were more likely to be found in small vessels and associated with increased adhesion, which could exacerbate vascular dysfunction (12). Peripheral neutrophils also express higher levels of CD11b in persons with AD, again suggesting the involvement of neutrophil adhesion in AD pathogenesis (56). Vascular dysfunction has been observed in AD patients before onset of cognitive dysfunction and has been associated with a reduced CBF, hypoperfusion, breakdown of endothelial tight junctions, neuronal death, and BBB immune cell infiltration including by neutrophils (15, 57, 58). Neutrophils outside of the brain may also contribute to AD pathology. Neutrophil-specific cytokines in cerebral spinal fluid associate with BBB impairment in persons with AD (59). Peripheral neutrophil-related soluble factors, including neutrophil gelatinase-associated lipocalin (NGAL), MPO, interleukin-8 (IL-8), and tumor necrosis factor (TFN), also associated with decline in executive function in patients with mild AD (25). These findings indicate that neutrophils are likely a significant contributor to increased neuroinflammation and BBB break down and potentially contribute to cognitive decline in AD patients.

Neutrophil function in AD remains understudied. The identification of NETs in brain tissue and NET markers in plasma in AD is suggestive that neutrophils may be prone to NET release (11, 53, 54). Peripheral blood neutrophils in persons with AD produce more ROS and neutrophil hyperactivation was associated with faster cognitive decline (53, 60). In addition, reduced neutrophil phagocytosis has been observed in patients with mild cognitive impairment and AD (61, 62). Another study demonstrated that neutrophil granule proteins bind Aβ and inhibit its aggregation (63). Additional beneficial roles for neutrophils in AD, including removal of Aβ, tissue repair, or immune system suppression remain unexamined. More studies illuminating neutrophil function and the mechanisms by which increased frequencies of activated neutrophils, or a loss of beneficial neutrophil functions may be involved in the plethora of pathophysiological processes associated with AD are needed.

## 3 Discussion

There is strong evidence that neutrophils infiltrate the brain during AD and multiple studies suggest mitigating neutrophil infiltration or targeting neutrophilic inflammation may be a beneficial therapeutic strategy (11, 12, 29). However, neutrophils are not a homogeneous population and the methods used to identify neutrophils in AD in studies published so far (Ly6G+ cells, MPO+ cells, GR-1+ cells) represent a population composed of cells with varying phenotypes and functions (7, 12). While multiple studies suggest that increased NETs may contribute to AD pathogenesis, it remains possible that neutrophils may be playing an anti-inflammatory role as gMDSCs, contributing in the removal of Aß *via* phagocytosis, or participating in tissue repair. Along these lines, one study demonstrated fewer low density neutrophils in persons with probable AD, which may represent fewer gMDSCs with immune suppressive properties (64). Another study demonstrated that reducing TNFα led to increased neutrophil infiltration, reduced pathogenic amyloid and tau accumulation, and improved cognitive performance (65). However, TNFα modulation may have shifted neutrophil functionality in addition to altering neutrophil frequency in the brain, but function was not examined. Future studies should focus on further investigating neutrophil functionality to determine their potential as a therapeutic target and investigate crosstalk with microglia and astrocytes or the various anti-inflammatory drugs already in the pipeline for AD that may alter neutrophil function or homeostasis.

Transcriptional and blood studies have demonstrated that age, sex, and *APOE* genotype are confounding variables that must be addressed in AD studies (25, 49, 59, 66, 67). Hypotheses for why AD is more common in females include sexual dimorphism of genes, hormones, and the immune system (66, 68, 69). Neutrophils also exhibit differences based on sex and their functionality decline with age (70, 71). In females, neutrophils have been shown to exhibit decreased NET release and reduced RNA expression of primary granules and elastase (45, 70) and increased MPO expression in comparison with their age matched male counterparts (69, 70). Several of these neutrophil factors, such as MPO and NET release have also been associated with an increased risk of AD (12, 25, 26, 45, 55). These data demonstrate the need for in-depth studies investigating the role of sex and age on neutrophil dysregulation in AD. Neutrophils should also be investigated in the context of genetic risk factors for AD. The *APOE4* allele, the greatest genetic risk factor for LOAD, is known to modulate inflammatory responses in microglia and astrocytes *via* inflammatory pathways important for neutrophil homeostasis and functionality (e.g. NFκb, MAPκ) (72). Thus, we hypothesize that the *APOE4* allele may increase inflammatory neutrophil responses. Neutrophils may also be impacted by microbiome alterations, which are well-established in AD (73–75). We have previously shown that microbiome alterations associate with increased neutrophil lifespan in chronic infection (36), and these connections should be investigated in AD to determine if microbiome modulation is a way to mitigate neutrophilic inflammation. Promoting neutrophil apoptosis is a potential way to promote the resolution of inflammation without severely compromising host defense (76), yet neutrophil apoptosis in AD has yet to be examined. Finally, further examination of neutrophil transcriptional changes or phenotypes may reveal additional biomarkers for early AD diagnosis, allowing for earlier AD treatment that delays progression.

## Author contributions

MLA drafted the article. THM conceptualized the article and assisted with writing and editing. All authors contributed to the article and approved the submitted version.
